# Inter-rater reliability and acceptance of the structured diagnostic interview for regulatory problems in infancy

**DOI:** 10.1186/s13034-016-0107-6

**Published:** 2016-07-05

**Authors:** Lukka Popp, Sabrina Fuths, Sabine Seehagen, Margarete Bolten, Mirja Gross-Hemmi, Dieter Wolke, Silvia Schneider

**Affiliations:** Clinical Child and Adolescent Psychology, Ruhr-Universität Bochum, Massenbergstraße 9-13, 44787 Bochum, Germany; Department of Developmental Psychopathology, Child and Adolescents Psychiatric Clinic, University Basel, Schanzenstrasse 13, 4056 Basel, Switzerland; Department of Psychology and Health Sciences Research Institute, Warwick Medical School, University of Warwick, Coventry, CV47AL UK; School of Psychology, University of Waikato, Private Bag 3105, Hamilton, 3240 New Zealand

**Keywords:** Regulatory problems, Baby-DIPS, Infancy, Structured diagnostic interview, Reliability, Acceptance

## Abstract

**Background:**

Regulatory problems such as excessive crying, sleeping–and feeding difficulties in infancy are some of the earliest precursors of later mental health difficulties emerging throughout the lifespan. In the present study, the inter-rater reliability and acceptance of a structured computer-assisted diagnostic interview for regulatory problems (Baby-DIPS) was investigated.

**Methods:**

Using a community sample, 132 mothers of infants aged between 3 and 18 months (mean age = 10 months) were interviewed with the Baby-DIPS regarding current and former (combined = lifetime) regulatory problems. Severity of the symptoms was also rated. The interviews were conducted face-to-face at a psychology department at the university (51.5 %), the mother’s home (23.5 %), or via telephone (25.0 %). Inter-rater reliability was assessed with Cohen’s kappa (*k*). A sample of 48 mothers and their interviewers filled in acceptance questionnaires after the interview.

**Results:**

Good to excellent inter-rater reliability on the levels of current and lifetime regulatory problems (*k* = 0.77–0.98) were found. High inter-rater agreement was also found for ratings of severity (*ICC* = 0.86–0.97). Participants and interviewers’ overall acceptance ratings of the computer-assisted interview were favourable. Acceptance scores did not differ between interviews that revealed one or more clinically relevant regulatory problem(s) compared to those that revealed no regulatory problems.

**Conclusions:**

The Baby-DIPS was found to be a reliable instrument for the assessment of current and lifetime problems in crying and sleeping behaviours. The computer-assisted version of the Baby-DIPS was well accepted by interviewers and mothers. The Baby-DIPS appears to be well-suited for research and clinical use to identify infant regulatory problems.

**Electronic supplementary material:**

The online version of this article (doi:10.1186/s13034-016-0107-6) contains supplementary material, which is available to authorized users.

## Background

For infants, major developmental tasks in the first months of life include adapting to the postnatal environment (e.g., to calm down when irritated), ingesting food and gaining weight and developing a sleep-wake-regulation. To master these tasks, infants rely on parental support to regulate their behavior [1–3]. If behavior regulation in infants does not develop appropriately, regulatory problems (RPs) in the form of excessive crying, feeding and sleeping difficulties can emerge as the earliest indicators of mental health difficulties in childhood.

Prevalence rates of RPs differ according to assessment method, age and definition. Recent studies have shown that approximately 12–25 % of infants in the first year of life are identified with sleeping problems [4], 16 % with excessive crying [5] and 1.5–3 % with feeding problems [6, 7]. Between 4 and 10 % of the infants show RPs in two of these areas [8]. About 1–2 % of 1-year-old infants exhibit all three problems simultaneously. This last group of infants is classified as suffering from a regulation disorder [5, 9].

Recent studies have shown that problems arising from RPs are not restricted to infancy. There are associations between RPs in infancy and emotional, behavioral and cognitive impairments in later childhood. In a meta-analysis including 22 studies conducted between 1987 and 2006, Hemmi and colleagues [10] found that children with RPs in infancy exhibited more behavioral problems, in particular externalizing problems, at later ages (age ranged between 1.3 and 10 years) compared to children without previous RPs. Further research indicated that the severity and number of early RPs predict unfavorable developmental outcomes such as delayed cognitive development and compromised social skills [9, 11]. Thus early detection of RPs during infancy appears to be crucial for preventing mental health issues and negative developmental outcomes in the long term.

For diagnosing RPs, a multi-method approach is recommended to obtain information about the infant’s behavior, the parent–child relationship and parental psychological strain [e.g., 1, 12–14]. Ideally, assessment of RPs includes a pediatric examination and structured observations of infant behavior with the help of a diary. Additionally, parent–child interactions ought to be evaluated live or from videotapes. Infant’s and parents’ mental health status should be assessed using questionnaires and diagnostic interviews [1].

Diagnostic interviews are the gold standard for detecting and differentiating clinically significant difficulties from symptoms that are not clinically relevant [15–17]. Yet, to our knowledge there are no structured diagnostic interviews available to assess RPs in the first year of life. Among other advantages, structured diagnostic interviews facilitate the exchange between the clinician and the caretaker and allow collecting relevant information within an acceptable time span [18, 19]. Having a reliable structured diagnostic interview for the assessment of RPs in infancy is therefore desirable.

In addition to the reliability and validity, a structured diagnostic interview must be feasible and therefore accepted by interviewers and interviewees to guarantee its use. Feasibility refers to how successful the implementation of the interview will be and acceptance is defined as the participants’ reaction to and in this case the evaluation of, the interview [20]. Studies with clinical and community samples of adults and children showed that structured diagnostic interviews for mental disorders are highly accepted across different clinical settings [21–25]. In contrast to the setting, the presence of mental disorders was found to influence the participants’ acceptance. Structured diagnostic interviews were rated less positively by adults and children with mental health disorders compared to participants without mental health problems [21]. The authors suggested that the referred participants felt more uncomfortable by talking about their problems and that the interviews took longer what might have been rated more negative than shorter interviews.

In the present study, the inter-rater reliability and acceptance of a structured computer-assisted diagnostic interview for regulatory problems (Baby-DIPS) was investigated. The interviewers and interviewees were asked to rate their acceptance of the computer-assisted Baby-DIPS [26] that was conducted at the mothers’ home or at a psychology department. Based on earlier findings [21–25], we expected comparable and high acceptance from interviewers and interviewed mothers across the two settings. We further investigated if the mothers’ acceptance of the Baby DIPS differed depending on the presence or absence of RPs in their infants. In line with previous studies we predicted that interviews that did not detect any RPs would be rated more positively by the participants compared to interviews that did indicate one or more RPs. In sum, the overall goal of the present study was to evaluate the (1) inter-rater reliability and (2) acceptance of the Baby DIPS in different settings (i.e., psychology department versus home) and as a function of infants’ diagnostic status (i.e., presence versus absence of any RPs).

## Methods

### Participants

The final sample consisted of *N* = 132 mothers. Interviews with six additional mothers were scheduled but could not be conducted due to the mothers cancelling their appointments without giving a reason. Data from this community sample were collected in the context of four different research studies at two sites, 87.9 % University of Basel, Switzerland and 12.1 % at Ruhr-Universität Bochum, Germany. Seventy-five percent were first-time mothers. The infants (50 % girls) were 10 months and 15 days old on average (range: 3;25–18;15). The majority of the German-speaking mothers had a Swiss (60.6 %) or a German nationality (37.1 %). The mothers’ mean age was *M* = 33.3 years (*SD* = 4.73) and the majority was highly educated (56.8 % had an A-Level) and lived in a relationship (98.5 %). Across studies, the participants were similar in terms of the infants’ gender (girls = 47.4–53.3 %) and mothers’ age (*M* = 32.9–34.0; *SD* = 4.1–5.3). Also, in all four studies more than 50 % of mothers reported an A-Level and more than 98 % were in a relationship with the biological father. There was a difference between the four studies regarding the infants’ age (*M* = 5.6–11.8 months; *SD* = 0.5–3.4 months).

The acceptance of the interview was assessed in one of the four research studies. Here, a questionnaire was completed by a sample of 48 mothers either at the mother’s homes (*n* = 17, 35.4 %) or at the psychology department of the University of Basel (*n* = 31, 64.6 %). Two additional data sets were excluded because fathers had completed the acceptance questionnaires. Characteristics of the group of mothers who completed the acceptance questionnaire were similar to those of the entire sample (*M*_*age*_ = 32.9 years, *SD* = 4.72; 52 % A-Level). Across participants, three interviewers completed the interviewer’s version of the acceptance questionnaire (interviewers’ mean age was *M* = 26.21, *SD* = 7.93).

### Participant recruitment and selection procedures

Mothers were recruited via personal contact, public health services, flyers, newspaper announcements, midwives, hospitals and gynecologists between February 2008 and June 2014. The Baby-DIPS interview was part of the regular assessment procedure for ongoing studies that had all been approved by the local ethical committees at the departments of Psychology of the University of Basel or Ruhr-Universität Bochum. To be included in the studies, mothers had to have an infant aged between 3 and 18 months without a diagnosed medical condition. Mothers were required to have a basic level of German literacy, allowing them to understand and respond to the Baby-DIPS interview questions.

### Measures and interviewers

#### The Baby-DIPS

The Baby-DIPS is a structured interview designed for the diagnosis of former and current RPs in infants and toddlers up to 3 years of age. Lifetime diagnoses are made by combining current and former diagnoses. Thus, they indicate whether RPs have existed at any time in the lifespan, including the present time. The Baby-DIPS is an adapted German version of the structured diagnostic interview “Parent Interview II” from the GAIN STUDY (Growth in At-risk Infants; [27]). The Parent-Interview II was translated into German and complemented in terms of content and structure. The main differences according to the diagnostic symptoms were the adaption of the Wessel’s rule for excessive crying and an age delimiter for the differentiation between sleep maintenance problems before and after the age of 6 months. Further questions (open and categorical) about typical thoughts, emotions and parenting behavior in the context of regulatory problems were added. Questions about the economic status, parent-infant attachment and life stressors were omitted.

The manual was additionally adapted to the well-established structure of the diagnostic interviews of the DIPS family [28, 29]. These structured diagnostic interviews are developed for the assessment for mental disorders according to DSM throughout the life span and based on the same underlying structure. The main characteristics that are also included in the Baby-DIPS are to skip rules for a more efficient implementation, the assessment of former diagnostic symptoms to consider lifetime diagnoses and the inclusion of a categorical (diagnoses) and dimensional (severity rating) coding system.

The Baby-DIPS assesses the clinical criteria of excessive crying according to the Wessel’s rule [30], feeding disorders according to DSM-IV-TR [31] and sleeping problems according to an adaption of the research diagnostic criteria for preschool-age (RDC-PA, [32] for an overview see Table 1). Furthermore, the Baby-DIPS includes comprehensive information on the different regulation problems allowing diagnoses of sleeping problems not only according to the above mentioned criteria sets but also to DC:0-3R [33] and RDC-PA [32]. Within the sleep category, two different problems are distinguished, a) settling at bedtime, b) sleeping through the night, plus the severe form of sleeping through the night. The existence of each problem results in the infant being diagnosed with an RP. Thus, an infant can be diagnosed with a maximum of four RPs in the Baby-DIPS (feeding, excessive crying and the two sleep problems). If all diagnostic criteria for a diagnosis are fulfilled the interviewer rates the severity of the symptoms on a scale from 0 (absent) to 8 (severe). A severity rating of four or higher indicates a clinically relevant diagnosis. Maternal settling behavior and related cognitions and emotions about the infants’ crying, feeding and sleeping behavior are additionally explored within the Baby-DIPS. Furthermore, descriptive information about the infant’s age, height, weight, siblings, medical history and complications during pregnancy are collected. The participant’s responses can either be recorded online (that is, computer-assisted) using a Microsoft Excel**©** spreadsheet or the protocol sheets can be printed out and filled in manually.

**Table 1:** Diagnostic criteria of regulatory problems assessed with the Baby-DIPS

Criteria	Excessive crying	Sleeping problems I (settling at bedtime)	Sleeping problems II (sleeping through the night)	Feeding problems
A	The child cries for more than three hours per day	The child needs more than one hour to fall asleep	The child is older than 6 months	Feeding disturbance as manifested by persistent failure to eat adequately with significant failure to gain weight or significant loss of weight over at least 1 month
B	The child cries for more than 3 days per week		The child awakes at least five times per week	The disturbance is not due to an associated gastrointestinal or other general medical condition (e.g. esophageal reflux)
C	The child cries for longer than 3 weeks		The child awakes at least once between 12 to 5 a.m.	The disturbance is not better accounted for by another mental disorder (e.g. rumination disorder) or by lack of available food
D			Severe form: The child awakes repeatedly per night	The onset is before age 6

#### Acceptance questionnaires

The acceptance questionnaires for participants and interviewers (see Additional file 1: Appendix S1 and Additional file 2: Appendix S2) were adapted from the acceptance questionnaires for structured diagnostic interviews for adults by Suppiger and colleagues [24]. The questions were rephrased for the use with parents of infants. The overall satisfaction with the interview was assessed on a scale from 0 (not at all satisfied) to 100 (completely satisfied). Additionally, statements about the interview content and the general procedure were rated on a 4-point Likert scale from 0 (disagree) to 3 (completely agree). Seven items were positively formulated and seven items were negatively formulated. At the end of the questionnaire there was space for comments. Questions about the use of a computer during the interview, the willingness to participate again and the recommendation of the interview were added to the acceptance questionnaire for the participants. Two questions regarding the use of a computer during the interview and the nature of questions were added for the interviewers. That is, interviewers rated if they felt the questions were too private or too detailed.

#### Interviewers

Across the entire sample, interviewers were 14 female postgraduate psychologists. They completed a standard training on the use of the Baby-DIPS. The training consisted of two steps. First, after the interview handbook was read and understood, the trainees rated two audiotaped interviews and matched their clinical decisions with the rating of their clinical supervisor. The aim was that the diagnoses and severity ratings were in agreement (±1 score). Second, the trainees conducted two audio-taped interviews with acquaintances that were compared to the coding of their clinical supervisor. The aim of the training was to achieve consistent diagnostic agreement on at least two interviews. Interviewers received regular group supervision as required to discuss questions, difficulties or diagnostic decisions.

### Procedure

Informed consent to participate in the respective study was given by all participants. An appointment for the Baby-DIPS was arranged on the phone. The mothers’ answers in the interviews were either manually recorded during the interview using a printed version of the Baby-DIPS (12 %) or during the interview on the computer. The interviews were conducted at the psychology department of the University of Basel (51.5 %), via telephone (25.0 %) or at the mothers’ home (23.5 %). All interviews were audio-taped so that a second blind rater could score the interview later to provide inter-rater reliability. The blind raters were Master students who received the standardized Baby-DIPS training described above. The acceptance questionnaires were completed after the interview by both the interviewer and the mother. The mothers who completed the questionnaire at home sent it back to University of Basel by mail. Mothers and infants who participated at the University of Basel received an age-appropriate toy for the infant to compensate for time and effort. The mothers who participated at Ruhr-Universität Bochum received a certificate about their participation in the research project and a colored picture frame.

### Analyses

All statistical analyses were conducted with SPSS 22.0 for Mac OS X. The coding and re-coding of every interview by two independent raters meant that two scores for each interview were available to determine inter-rater reliability. Inter-rater agreement of diagnoses were determined with Kappa values (*k*) [34], with *k* < 0.4 indicating poor, 0.4 to 0.6 moderate, 0.6 to 0.8 good and >0.8 excellent agreement [35]. Statistical significance of the kappa coefficient was determined with χ^2^-exact tests. The Kappa coefficient is a standard measurement for the analysis of agreement on a binary outcome between two raters but it is often criticized for its dependence on the observed prevalence [36]. For this reason, kappa values are reported for diagnoses with a minimum base rate of ten percent [37, 38]. Furthermore, the percentage of total agreement and Yule’s Y [39] as a chance-corrected, base-rate independent measure of agreement was calculated for reasons of comparison [40]. The values of Yules Y range from −1 to 1 implying perfect negative or positive agreement. Standards for the interpretability are not established [41]. Inter-rater agreement of the severity ratings was evaluated by calculating the intra-class correlation coefficients (ICC) as a measure of reliability of continuous data [41]. ICC’s range from −1 to 1 and are interpreted as <0.20 poor, 0.30–0.40 fair, 0.50–0.60 moderate, 0.70–0.80 strong and >0.80 almost perfect agreement [42, 43].

The patients’ and interviewers’ acceptance of the Baby-DIPS was explored with descriptive measures. T-tests for independent samples were conducted to explore differences in the satisfaction with the interview between mothers who were interviewed at home versus at the psychology department of the University of Basel and between mothers whose infants met at least one RP versus no problems.

## Results

The interviews had a mean duration of *M* = 43.79 (*SD* = 13.95, Range 14–91) min. Seventy (53 %) infants of the interviewed mothers met diagnostic criteria for at least one RP (lifetime diagnoses). Frequencies of diagnoses are shown in Table 2.

**Table 2:** Number (%) of current and lifetime regulatory problems according to the original interview data (rater 1)

	Regulatory problems (%)				
	Feeding	Sleeping I (settling at bedtime)	Sleeping II (through the night)	Severe form of sleeping II	Excessive crying
Current	1 (0.76)	8 (6.1)	26 (19.7)	19 (14.4)	2 (1.5)
Lifetime	2 (1.5)	22 (16.7)	42 (31.8)	31 (23.5)	27 (20.5)

Of the displayed data, infants met criteria for comorbid diagnoses with two (current: 17, lifetime: 29), three (current: 2, lifetime: 11) and four (current: 0, lifetime: 1) diagnoses. Every infant who met the criteria for the severe form of sleeping problems met also the criteria for the not severe form of sleeping problems

Inter-rater reliability data is presented in Table 3. Overall, good to excellent inter-rater concordance on the Baby-DIPS diagnoses was found with kappa values of current (*k* = 0.77–0.85) and lifetime diagnoses (*k* = *0*.83–0.98). The raters also showed excellent agreement on the decision not to give a current (*k* = *0.*80) or lifetime (*k* = 0.92) diagnosis. Kappa values could not be calculated for all RPs with a lower base rates than 10 %.

**Table 3:** Inter-rater agreement on regulatory problems assessed with The Baby-DIPS (N = 132)

Regulatory problems	Frequencies (Rater 1/Rater 2) −/− −/+ +/− +/+		Estimated prevalence (%)	Total agreement (%)	Cohen’s kappa (*SE*)	Yule’s Y	ICC (95 % CI)
Current feeding	130	1	1.5 (1.14)	99.2	–	–	0.41 (0.26–0.54)
	0	1					
Sleeping I (settling at bedtime)	121	3	8 (6.06)	95.5	0.60 (0.15)*	0.78	0.73 (0.64–0.80)
	3	5					
Sleeping II (throught the night)	105	1	24 (18.18)	95.5	0.85 (0.06)***	0.91	0.90 (0.86–0.93)
	5	1					
Severe form of sleeping II	111	2	17.5 (13.26)	94.7	0.77(0.08)***	0.85	0.86 (0.80–0.90)
	5	5					
Excessive crying	130	0	2 (1.52)	100	–	–	1.0
	0	2					
No diagnosis	28	7	99 (75.0)	92.4	0.80 (0.06)***	0.84	–
	3	94					
Lifetime feeding	129	1	2.5 (1.89)	99.2	0.80*** (0–3 × 10^−4^)	–	0.67 (0.56–0.75)
	0	2					
Sleeping I (settling at bedtime)	108	2	21 (15.91)	95.5	0.83 (0–3 × 10^−4^)***	0.88	0.92 (0.89–0.94)
	4	18					
Sleeping II (throught the night)	87	3	42.5 (32.2)	96.2	0.91 (0–3 × 10^−4^)***	0.92	0.95 (0.94–0.97)
	2	40					
Severe form of sleeping II	97	4	31.5 (23.86)	94.7	0.85 (0–3 × 10^−4^)***	0.88	0.93 (0.90–0.95)
	3	28					
Excessive crying	106	0	26.5 (20.08)	100	0.98 (0–3 × 10^−4^)***	–	0.97 (0.95–0.98)
	1	26					
No diagnosis	65	5	64.5 (48.86)	96.2	0.92 (0–3 × 10^−4^)***	–	–
	0	62					

Where estimated prevalences do not equal or exceed 10 of the total observations (displayed in parentheses), kappa coefficients may underestimate agreement. Kappa coefficients are not calculated if no disorder is identified by at least one rater. Yule’s Y coefficients are incalculable if either cell frequency of the contingency tables equals zero. Significance of the kappa coefficients was determined with χ^2^-exact tests. Intra class coefficients (ICC) were calculated with a two-way mixed model, interpreting the single measure of the coefficients. Significance of the intra-class coefficients was detected with *F*-tests

* p < 0.05; ** p < 0.01; *** p < 0.001

The intra-class correlation coefficients showed strong to almost perfect agreement on the severity of current (0.86–0.90) and lifetime (0.92–0.97) diagnoses.

A total of 48 mothers completed the acceptance questionnaire about the computer-assisted version of the Baby-DIPS. Four mothers and two interviewers did not complete the scale measuring overall satisfaction but all other questions. The mothers’ overall mean satisfaction rating with the interview was 88.57 (*SD* = 11.03) with a range from 60 to 100. The mothers reported high acceptance of the Baby-DIPS over all items and in different settings (see Table 4). An independent-samples *t* test showed no significant difference in the mean scores of the overall satisfaction with the interview between settings (i.e., home or at the psychology department of the University of Basel), *t*(42) = 1.45, *p* = 0.16. Likewise, there was no significant difference in acceptance ratings between the mothers of infants with versus without an RP, *t*(42) = 1.51, *p* = 0.14.

**Table 4:** Means (*SD*) for the acceptance questionnaires for participants and interviewers for different settings and presence of regulatory problems

Item no.	Item	All participants (*N* = 48)	Setting I (at home; *n* = 31)	Setting II (at University; *n* = 17)	Regulatory problem present (*n* = 32)	Regulatory problem absent (*n* = 16)
*Acceptance questionnaire for participants*						
	Overall satisfaction^1^	88.57 (11.03)	90.40 (10.71)	85.53 (11.17)	86.90 (11.02)	92.14 (10.57)
1	Felt comfortable	2.73 (0.57)	2.74 (0.63)	2.71 (0.47)	2.63 (0.66)	2.94 (0.25)
2	Computer scared me	2.94 (0.25)	2.97 (0.18)	2.88 (0.33)	2.91 (0.30)	3.0 (0)
3	Would participate again	2.79 (0.46)	2.84 (0.37)	2.71 (0.59)	2.75 (0.51)	2.87 (0.34)
4	Wished to cancel	2.85 (0.55)	2.81 (0.65)	2.94 (0.24)	2.87 (0.42)	2.81 (0.75)
5	More confused	2.96 (0.20)	2.94 (0.25)	3.0 (0)	2.97 (0.18)	2.94 (0.25)
6	Questions too private	2.85 (0.51)	2.87 (0.34)	2.82 (0.73)	2.81 (0.59)	2.94 (0.25)
7	Recommend participation	2.50 (0.65)	2.55 (0.57)	2.41 (0.80)	2.41 (0.71)	2.69 (0.48)
8	Positive relationship	2.81 (0.64)	2.74 (0.77)	2.94 (0.24)	2.84 (0.57)	2.75 (0.78)
9	Exhausting	2.77 (0.59)	2.81 (0.48)	2.71 (0.77)	2.69 (0.69)	2.94 (0.25)
10	Felt well-understood	2.54 (0.58)	2.61 (0.56)	2.41 (0.62)	2.47 (0.62)	2.69 (0.48)
11	Detailed questioning	2.43 (0.68)	2.5 (0.63)	2.29 (0.77)	2.25 (0.72)	2.80 (41)
12	Typing was annoying	2.85 (0.62)	3.0 (0)	2.59 (1.0)	2.78 (0.75)	3.0 (0)
13	Felt questioned	2.79 (0.58)	2.71 (0.69)	2.94 (0.24)	2.87 (0.34)	2.63 (0.89)
14	Better understanding	0.25 (0.64)	0.23 (0.67)	0.29 (0.59)	0.22 (0.61)	0.31 (0.70)
*Acceptance questionnaire for interviewers*						
	Overall satisfaction^1^	85.37 (13.97)	85.62 (11.14)	84.94 (18.20)	84.97 (15.96)	86.29 (8.18)
1	Conducted in all conscience	2.52 (0.55)	2.55 (0.57)	2.47 (0.51)	2.53 (0.57)	2.5 (0.52)
2	Mistakes	2.67 (0.52)	2.65 (0.49)	2.71 (5.9)	2.72 (0.52)	2.56 (0.51)
3	Exhausting	2.58 (0.71)	2.68 (0.60)	2.41 (0.87)	2.56 (0.76)	2.62 (0.62)
4	Questions too detailed	2.65 (0.64)	2.55 (0.72)	2.82 (0.34)	2.63 (0.71)	2.69 (0.48)
5	Extensive information	2.56 (0.54)	2.58 (0.56)	2.53 (5.1)	2.62 (0.55)	2.44 (0.51)
6	Typing was annoying	2.56 (0.62)	2.71 (0.53)	2.29 (6.9)	2.47 (0.67)	2.75 (0.45)
7	Computer scared me	3.0 (0)	3.0 (0)	3.0 (0)	3.0 (0)	3.0 (0)
8	Questions too private	2.88 (0.33)	2.90 (3.0)	2.82 (0.39)	2.87 (0.34)	2.88 (0.34)
9	Did not report everything	2.33 (1.0)	2.26 (1.10)	2.47 (0.87)	2.44 (0.95)	2.13 (1.15)
10	Differentiated perception	2.44 (0.62)	2.39 (0.67)	2.53 (0.51)	2.50 (0.62)	2.31 (0.60)
11	Positive relationship	2.44 (0.58)	2.48 (0.51)	2.35 (0.70)	2.56 (0.50)	2.44 (0.51)
12	Participant’s cooperation	2.46 (0.62)	2.19 (0.65)	2.71 (0.47)	2.16 (0.72)	2.25 (0.76)
13	Empathy	2.15 (0.71)	2.19 (0.65)	2.06 (0.83)		2.13 (0.72)

Overall satisfaction rated on scale of 0 to 100 (0 = not at all satisfied, 100 = totally satisfied); all other items rated on a scale of 0 to 3 (0 = disagree, 1 = slightly agree, 2 = almost completely agree, 3 = completely agree); Items 1–10 are given in full in Additional file 1: Appendix S1. Items 2, 4–6, 9, 12 and 13 were negatively formulated in the participants’ version and items 2–4 and 6–9 in the interviewer’s version. Negative formulated items were reversed so that a higher number means less agreement with the negative statement and higher satisfaction

^1^Four participants and two interviewers did not filled in the scale measuring the overall satisfaction

The mean interviewer rating in terms of overall satisfaction with the interview was *M* = 85.37 (*SD* = 13.97), ranging from 30 to 100 (Table 4). Independent-samples t-tests revealed no significant differences in overall satisfaction scores between settings [*t*(44) = 0.14, *p* = 0.89] or infants who had versus did not have RPs [*t*(44) = 0.37, *p* = 0.71].

## Discussion

The present findings indicate that the Baby-DIPS is a reliable and acceptable structured diagnostic interview for the assessment of RPs in infancy. Overall, inter-rater reliability was good to excellent for current and lifetime RPs. Importantly, a high inter-rater agreement was also found for the absence of RPs. Similarly, a strong agreement between the raters on the severity ratings of assessed RPs was found. It should be mentioned that the inter-rater reliability was not assessed for feeding difficulties due to a low base rate (see Table 3). These findings cannot be compared to other interviews for RPs in infancy because the Baby-DIPS is the first structured diagnostic interview specifically for RPs adaptable to the first year of life. The Baby-DIPS showed similar levels of inter-rater agreement as the parent-version of the Kinder-DIPS [37], which has good inter-rater agreement on lifetime major diagnostic categories (*k* = 0.94–0.97).

Furthermore, the acceptance of interviewers and interviewees with the computer-assisted Baby-DIPS was assessed in the present study. The overall average satisfaction score with the interview was high for interviewers and participants across different settings indicating that the Baby-DIPS was well accepted for diagnostic purposes both at the participants’ home and at the psychology department of the University of Basel. These data are in line with previous studies showing that across different settings, structured diagnostic interviews are generally highly accepted and appreciated by participants and clinicians who are experienced with structured interviews [21, 22, 24]. Aspects of the interview that were rated particularly favourably by participants and interviewers were the number and type of questions, use of a computer during the diagnostic process and the relationship between interviewer and interviewee.

The overall positive acceptance rating from interviewers and participants supports the view that potential concerns of therapists about patients feeling interrogated through the interview or that patients might perceive the relationship with the interviewer as negative during a diagnostic interview are unfounded [44].

### Limitations and future directions

Several limitations of this study should be mentioned. First, other psychometric properties as the test-re-test reliability and the validity of the Baby-DIPS have not been assessed yet. Further investigation of these properties will be valuable to ensure that the Baby-DIPS consistently measures what it was designed to assess. Here, two major challenges could emerge: (1) Test-re-test reliability might well be influenced by infants’ rapid development. In our view, a re-assessment using the Baby-DIPS should occur within 4 weeks of the first interview (2). Diagnostic interviews have rarely been validated so far. This is likely due to a lack of an external criterion. Until now, there is no assessment available that could be regarded as a gold standard or irrevocable truth for identifying RPs. The ratings of specific criteria always result from the interview and have not been obtained beforehand with an objective measure to check the sensitivity and specificity of the assessment [45]. Nevertheless, a valuable approach might be to assess concordant validity of the Baby-DIPS with other assessment methods [46]. Here, different methods that assess crying, feeding and sleeping habits as questionnaires, diaries or psychophysiological measurements (e.g., sleep EEG) might confirm the validity of the Baby-DIPS diagnostic criteria. When this has been done, high agreement between measures and interview have been found [47, 48].

Second, the present sample is not representative with regard to socio-demographic status of the population of mothers and fathers with babies since it includes an unselected community sample of predominantly first-time mothers. Thus, future studies with larger sample sizes are needed to test for age effects on inter-rater reliability. The investigation of the inter-rater reliability in selected population-like samples with high neonatal risk factors, such as preterm birth or maternal depression would furthermore be of value.

In addition, only mothers were interviewed in the present study whereas in clinical practice, the mother, the father or both parents can be interview partners. The investigation of the psychometric properties of the Baby-DIPS and the acceptance of the interview with fathers and couples would therefore give a more complete picture of the clinical usability of the Baby-DIPS. Finally, the sample of mothers who completed the acceptance questionnaire was small. The generalizability of the acceptance outcomes should therefore be investigated in future studies with a larger sample size.

Third, the diagnostic criteria for RPs are constantly changing due to revisions of the major classification systems such as the DSM-5 [33] and guidelines for RPs in infancy (e.g., Zero to Three, [33]). The use of the diagnostic criteria for sleeping problems provided by Wolke [49] might have led to an overestimation of the prevalence of sleeping problems in the current sample. One possible explanation might be that Wolke provided an earlier age of onset (6 vs. 12 months) than the DC: 0-3R guidelines (12 months; awaken >30 min) (2005). The age delimiter of 6-respectively 12 months of age is still debated. The age delimiter of 6 months were used in this study because current research showed that infants are in state resettle themselves without parental support in the first three month of age [50]. Additionally, the criterion of how long a child must be awake at night to fulfill the criterion is different between the Baby-DIPS (asking for attention until parents come) and other criteria sets [32, 33] (awaken >30 min.) and thus leading to different prevalence rates. More empirical data is therefore needed to validate the current diagnostic criteria. Nevertheless, the Baby-DIPS must be regularly adapted to the latest versions of the common diagnostic guidelines since the reliability of a diagnostic interview in particular depends on the sensitivity of the underlying classification system to differentiate clinical significant from non-significant diagnostic criteria [51].

Finally, coefficients for the inter-rater reliability could not be examined for RPs with a lower prevalence rate of 10 because the base rate dependency of kappa coefficients might lead to an underestimation of the inter-rater concordance [40]. In the present study this was the case for feeding problems and current excessive crying. Inter-rater reliability must be therefore investigated in future studies with a larger or a clinical sample that comprises higher numbers of feeding problems and current excessive crying.

## Conclusion

The present findings support that the Baby-DIPS is a reliable instrument to assess excessive crying and sleeping problems in infants. The interviewers and participants showed high acceptance of the computer-assisted interview across different settings unrelated to the existence of RPs, indicating that the interview is feasible in the clinical practice. The present findings are to be complemented by the evaluations of the test re-test reliability and the validity of the Baby-DIPS.

## Additional files

10.1186/s13034-016-0107-6 Participant acceptance questionnaire.
10.1186/s13034-016-0107-6 Interviewer acceptance questionnaire.

## Abbreviations

RP: regulatory problem
DIPS: diagnostisches interview für psychische störungen (diagnostic interview for mental health problems)
Baby-DIPS: diagnostisches interview für regulationsprobleme im säuglings–und kleinkindalter (diagnostic interview for regulatory probelms in infancy)
