# Supplementary material for: Inter-rater reliability and acceptance of the structured diagnostic interview for regulatory problems in infancy
Source: Child Adolesc Psychiatry Ment Health. 2016 Jul 5;10:21. doi: 10.1186/s13034-016-0107-6 (PMC4932761; doi:10.1186/s13034-016-0107-6)
Supplement: Supplementary file 1 — 10.1186/s13034-016-0107-6 Participant acceptance questionnaire. [file 13034_2016_107_MOESM1_ESM.docx]

Appendix 1: Participant Acceptance Questionnaire

The participant acceptance questionnaire assessed global satisfaction with the structured interview (rating scale from 0=not at all satisfied to 100=totally satisfied). Additional 13 items measured the participant’s acceptance of structured interviews (4-point-Likert-type scale ranging from 0=disagree to 3=completely agree).

| 1. | I felt the interview was pleasant | (Ich empfand das Interview als angenehm) |
| --- | --- | --- |
| 2. | Anything that has to do with computers, makes me uncomfortable or scares me | (Alles was mit Computern zu tun hat, gibt mir ein unangenehmes Gefühl oder macht mir Angst) |
| 3. | I would participate again. | (Ich würde wieder mitmachen) |
| 4. | In between, I would have liked to break up the interview | (Zwischendurch hätte ich das Interview gerne abgebrochen) |
| 5. | I feel more confused than before the interview | (Ich fühle mich verwirrter als vor dem Interview) |
| 6. | The questions were too personal. | (Die gestellten Fragen waren zu persönlich) |
| 7. | I would recommend the interview to others | (Ich würde das Interview weiterempfehlen) |
| 8. | The relationship to my interviewer was positive | (Die Beziehung zum Interviewer habe ich als angenehm empfunden) |
| 9. | The interview was exhausting | (Das Interview war zu anstrengend) |
| 10. | I think the interviewer asked for enough detail to get an appropriate understanding of my situation | (Ich habe das Gefühl, der Interviewer hat genau nachgefragt, um meine Situation zu verstehen) |
| 11. | I think it’s good that the interview was conducted in such exact and detailled way | (Ich finde es gut, dass die Befragung so exakt und detailliert durchgeführt wurde) |
| 12. | I found it disturbing that the interviewer kept typing something into the computer | (Ich fand es störend, dass der/die Interviewer/in immer wieder etwas in den Computer eintippte) |
| 13. | I feel „questioned out“ | (Ich fühle mich ausgefragt) |
| 14. | I feel that I can understand my child better after the interview | (Ich habe das Gefühl, dass ich mein Kind nach dem Interview besser verstehe) |
