# Supplementary material for: Inter-rater reliability and acceptance of the structured diagnostic interview for regulatory problems in infancy
Source: Child Adolesc Psychiatry Ment Health. 2016 Jul 5;10:21. doi: 10.1186/s13034-016-0107-6 (PMC4932761; doi:10.1186/s13034-016-0107-6)
Supplement: Supplementary file 2 — 10.1186/s13034-016-0107-6 Interviewer acceptance questionnaire. [file 13034_2016_107_MOESM2_ESM.docx]

Appendix 2: Interviewer Acceptance Questionnaire

The interviewer acceptance questionnaire assessed global satisfaction with the structured interview (rating scale from 0=not at all satisfied to 100=totally satisfied). Additional 13 items measured the interviewer’s acceptance of structured interviews (4-point-Likert-type scale ranging from 0=disagree to 3=completely agree).

| 1. | I conducted the interview as well as I could | (Ich habe das Interview nach bestem Wissen und Können geführt) |
| --- | --- | --- |
| 2. | I made mistakes in administering the interview | (Bei der Interviewführung sind mir Fehler unterlaufen) |
| 3. | The interview was exhausting | (Das Interview war zu anstrengend) |
| 4. | I felt uncomfortable asking such exact and detailled questions. | (Ich fand es unangenehm so exakte und detaillierte Fragen zu stellen) |
| 5. | I feel that I got all the important information on the baby’s feeding, sleeping and crying behavior. | (Ich habe das Gefühl alle wichtigen Informationen über das Fütter-, Schlaf- und Schreiverhalten des Babys erhalten zu haben) |
| 6. | I felt uncomfortable having to keep typing something into my computer during the interview. | (Ich fand es unangenehm, während des Interviews immer wieder etwas in meinen Computer einzutippen) |
| 7. | Anything that has to do with computers, makes me uncomfortable or scares me. | (Alles was mit Computern zu tun hat, gibt mir ein unangenehmes Gefühl oder macht mir Angst) |
| 8. | I feel that the questions were too personal for the participant. | (Ich habe das Gefühl, die Fragen waren zu persönlich für den/die Teilnehmer/in) |
| 9. | I think the participant did not report everything that was bothering him/her | (Ich denke, der/die Teilnehmer/in hat nicht alles hervorgebracht, was sie/ihn bewegte) |
| 10. | The participant perceives himself/herself and his/her problems in a differentiated manner | (Der/Die Teilnehmer/in nimmt sich und seine Situation / Probleme differenziert wahr) |
| 11. | The relationship to the participant was positive | (Die Beziehung zum/r Teilnehmer/in habe ich als angenehm empfunden) |
| 12. | During the interview I experienced the participant as cooperative | (Ich erlebte den/die Teilnehmer/in im Gespräch als kooperativ) |
| 13. | I succeeded in being responsive to the participant | (Es ist mir gelungen auf den/die Teilnehmer/in einzugehen) |
